# Selection for Favorable Health Traits: A Potential Approach to Cope with Diseases in Farm Animals

**DOI:** 10.3390/ani10091717

**Published:** 2020-09-22

**Authors:** Guoyu Hu, Duy Ngoc Do, Janine Gray, Younes Miar

**Affiliations:** Department of Animal Science and Aquaculture, Dalhousie University, Truro, NS B2N 5E3, Canada; gy494270@dal.ca (G.H.); duy.do@dal.ca (D.N.D.); Janine.gray@dal.ca (J.G.)

**Keywords:** diseases, farm animals, genetic selection, health traits

## Abstract

**Simple Summary:**

The losses caused by the outbreak of diseases are disastrous for the animal farming industries. There is an urgent need for an efficient, economical, and permanent disease control method to cope with the adverse effects of diseases in farm animals. In this review, we have proposed that genetic/genomic selection for animals with favorable health traits provide potential methods to eliminate the adverse influences of diseases in farm animals. It is undeniable that the traditional methods for disease control (e.g., vaccination, treatment, and eradication strategy) and several other rising disease control and detection methods (e.g., genome editing, biosensor, and probiotics) are contributing to the prevention of diseases from farm animals, curing infected animals, and detecting sick individuals; however, the limitations and deficiencies of these methods cannot be ignored. Although genetic/genomic selection solutions are facing some challenges, the developments of selection-associated techniques (e.g., high throughput phenotyping and sequencing, and generation of big data) and the advantages of selection over the other disease control methods can provide animal farming industries the ability to cope with the issues caused by diseases through breeding for health traits.

**Abstract:**

Disease is a global problem for animal farming industries causing tremendous economic losses (>USD 220 billion over the last decade) and serious animal welfare issues. The limitations and deficiencies of current non-selection disease control methods (e.g., vaccination, treatment, eradication strategy, genome editing, and probiotics) make it difficult to effectively, economically, and permanently eliminate the adverse influences of disease in the farm animals. These limitations and deficiencies drive animal breeders to be more concerned and committed to dealing with health problems in farm animals by selecting animals with favorable health traits. Both genetic selection and genomic selection contribute to improving the health of farm animals by selecting certain health traits (e.g., disease tolerance, disease resistance, and immune response), although both of them face some challenges. The objective of this review was to comprehensively review the potential of selecting health traits in coping with issues caused by diseases in farm animals. Within this review, we highlighted that selecting health traits can be applied as a method of disease control to help animal agriculture industries to cope with the adverse influences caused by diseases in farm animals. Certainly, the genetic/genomic selection solution cannot solve all the disease problems in farm animals. Therefore, management, vaccination, culling, medical treatment, and other measures must accompany selection solution to reduce the adverse impact of farm animal diseases on profitability and animal welfare.

## 1. Introduction

Disease control is a global challenge for livestock industries and farmers, as diseases bring tremendous economic losses to farm animal production systems. The animal farming systems in both developed and developing countries are suffering economically from different infectious diseases. Direct economic losses from the outbreaks of disease can account for up to 20% of the revenue in developed countries and up to 50% of the revenue within the livestock sector of the developing world [1]. Basically, all farm animal production systems are vulnerable to disease. Many diseases, such as bovine viral diarrhea (BVD), Johne’s disease, and bovine respiratory disease complex (BRDC) in cattle farming; bluetongue and sheep pox in sheep farming; porcine reproductive and respiratory syndrome (PRRS), and African swine fever (ASF) in the swine industry; Newcastle disease and Marek’s disease in the poultry industry; and Aleutian disease in the mink industry, contribute to economic losses and cause serious animal welfare issues via persistent infection, increased mortality, reduced productivity and reproduction performance, and decreased product quality. Therefore, finding the effective solutions to combat diseases has become a top priority for all livestock industries.

To control diseases, many methods have been used with some level of success. Vaccination, medical treatment, and eradication strategy are common methods to control health issues caused by diseases. These methods, however, are facing some bottlenecks, such as the side effects of vaccination [2,3], public concerns about residual drugs and drug resistance after employing medical treatment [4,5,6,7,8,9], and financial cost and high recurrence rate of using eradication strategies [10,11]. Several other methods including genome editing, biosensor, and probiotics provide animal farming industries more options to enhance animal health. Unfortunately, the lack of effective legal oversight (e.g., genome editing) and technological immaturity (e.g., genome editing, probiotics, and biosensor) make these technologies not widely available for controlling diseases of farm animals. This makes seeking alternative solutions one of the main concerns for animal producers.

Breeding for favorable health traits is one solution that is highly anticipated. Health traits mainly include health body traits, disease susceptibility traits, and immune system traits. Selecting favorable health traits, which are complex traits influenced by many genes and environmental factors is a powerful tool against disease [12]. Host genetics is significant in controlling the health status of each individual in the same environment. Compared with the other methods of disease control in farm animals, the selection of animals with favorable health traits such as disease resistance, disease tolerance [13], and immunity responses [14] has many advantages. Classical genetic selection and genomic selection are playing important roles in genetically improving health and controlling diseases. Although many challenges exist in both selection methods, the great potential to genetically eradicate diseases from farming systems is still attracting the attention of many animal farming industries.

Given the importance of disease in farm animals and the dramatic development of technologies for disease characterization, it is crucial to have a comprehensive and holistic view about challenges and solutions for combating disease in farm animals. Therefore, this review paper was written: (1) to present an overview of common diseases in farm animals and the methods used to control them; (2) to highlight the advantages of coping with diseases by selecting for health traits through genetic or genomic selection, as well as the current stages of selection on major diseases in livestock industries; and (3) to discuss the major challenges of employing health trait selection and the potential solutions that can help improve selection.

## 2. Farm Animal Diseases: Influence, Prevalence, and Controlling Issues

### 2.1. The Influence, Prevalence, and Controlling Issues of Common Diseases in Farm Animals

**Figure 1:**
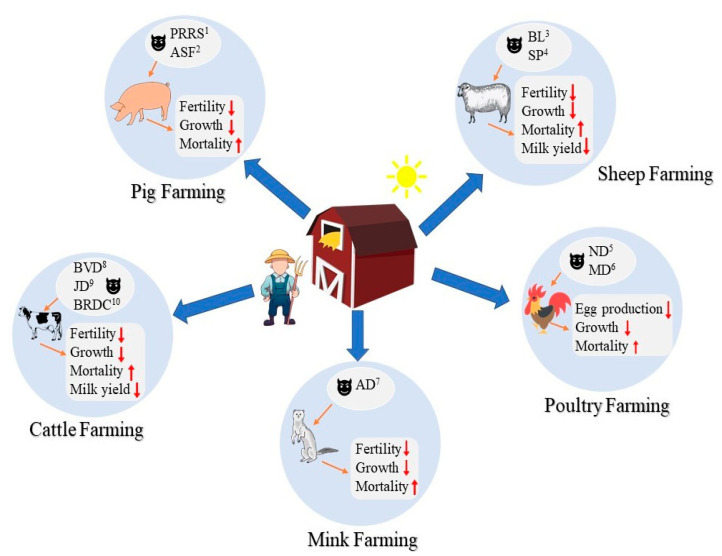
Economic consequences of common diseases in farm animals including pig, sheep, poultry, mink, and cattle. The upward-pointing arrows refer to increase, and the downward-pointing arrows refer to decrease (PRRS^1^ = Porcine reproductive and respiratory syndrome; ASF^2^ = African swine fever; BL^3^ = Bluetongue; SP^4^ = Sheep pox; ND^5^ = Newcastle disease; MD^6^ = Marek’s disease; AD^7^ = Aleutian disease; BVD^8^ = Bovine viral diarrhea; JD^9^ = Johne’s disease; BRDC^10^ = Bovine respiratory disease complex).

Disease in farm animals is a significant challenge to farm animal industries worldwide. Cattle, sheep, swine, poultry, and fur-bearing animals such as mink are the most important farm animals for human society and provide the main resource of milk, meat, egg, wool, and fur. Unfortunately, all these important farming systems are vulnerable to disease (Figure 1).

In cattle, BVD, Johne’s Disease, and BRDC are the most costly and persistent diseases (Table 1). The BVD commonly causes respiratory and reproductive complications in the herd. The prevalence of BVD in Northern Ireland can reach as high as 98.5% in non-vaccinated dairy herds and 98.3% in beef herds [15]. The BVD causes the dairy industry to lose 40 to 100 thousand US dollars per herd in Canada and 10 to 40 million US dollars per million calvings in Europe [16,17]. Culling infected animals and vaccinations are employed as short-term strategies to control this disease; however, they do not effectively eradicate BVD from the dairy farms [18,19]. Johne’s disease affects the small intestine of ruminant animals and results in weight loss, diarrhea, decreased fertility, and death. The current strategy of controlling Johne’s disease is based on timely detection through *Mycobacterium avium* ssp. *Paratuberculosis* enzyme-linked immunosorbent assay testing and then culling infected animals as there is no effective vaccine or treatment. For this reason, Johne’s disease is still rampant worldwide [10]. Approximately 68% of dairy operations in the USA were affected by this disease [20]. This disease causes economic losses of 15 million Canadian dollars per year to the dairy industries in Canada, and 200 to 250 million US dollars per year in the USA [21]. The BRDC, which is usually associated with infections of the lungs, causes pneumonia in calves and has been regarded as one of the primary causes of morbidity and mortality in beef farming [22,23]. In the USA, BRDC is the leading natural cause of death in beef cattle and causes financial losses of more than one billion US dollars annually [24]. The main method of controlling BRDC is using antibiotics; however, bacterial pathogen resistance to antibiotics for BRDC has caused the producers, practitioners, and the animal health industry to doubt the sustainability of using antibiotics to control BRDC [25].

In sheep, bluetongue and sheep pox are two common diseases in the sheep industry, causing significant economic losses (Table 1). Bluetongue causes huge economic losses to the sheep industry due to high mortality and morbidity, as well as the trading of animals associated with its outbreak. The prevalence of bluetongue was 19% in Italy [26], but in Sudan, the prevalence has been as high as 94% [27]. In 2007, the cost of the bluetongue disease for sheep breeding farms in the Netherlands was estimated at 12.6 million euros [28]. Vaccination has been regarded as the most viable method for the prevention and eradication of bluetongue disease; however, the expensive cost and potential side effects seriously influence the practicality and effectiveness of bluetongue disease vaccine [29]. Sheep pox is a serious, and often fatal infectious disease in sheep and causes a high mortality rate in sheep populations. Although live vaccines have been developed and are used worldwide, sheep pox still persists in regions where vaccination is routinely practiced, causing huge economic losses to the sheep industry [30]. Up to 22% [31] and 40% [32] of sheep in India and Ethiopia were infected by this disease, respectively. Annual economic losses from sheep pox disease in Maharashtra, India, were 2.4 million US dollars due to high mortality rates [33].

In swine, outbreaks of contagious diseases, such as PRRS and ASF, have not only resulted in significant economic losses for swine industries but have also caused animal welfare and environmental concerns (Table 1). The PRRS can cause anorexia, lethargy, hyperemia of the skin, dyspnea, hyperthermia, increased mortality rates, and reduction in average daily gain [34]. Up to 48% of swine farms in Ontario, Canada, were infected by PRRS from 2010 to 2013 [35]. In 2013, the total annual losses due to PRRS in the US were estimated at 664 million US dollars [36]. In Canada, the cost of PRRS was estimated at 130 million Canadian dollars per year [37]. Vaccination is considered the most feasible method for PRRS control; however, the high mutation rate and antigenic variability of the PRRS virus influences the effectiveness of controlling PRRS through vaccination. Meanwhile, the limited protection period of the vaccine against PRRS makes vaccination effective for only short time periods instead of eradicating the virus permanently [38,39]. The ASF is a viral disease that leads to high morbidity and mortality in swine and has drastic influences on global domestic swine production. The absence of an effective vaccine and available methods of disease control causes tremendous economic losses to the infected areas [40]. The ASF was reported in most provinces of China from August 2018 to July 2019 and resulted in an insufficient supply of pork products in China. The overall mean rate of incidence was 12.5%, and the highest incidence rate of 30% occurred in April–May 2019 [41]. In Russia, ASF has resulted in the loss of 800,000 pigs and 0.83–1.25 billion US dollars since its outbreak in 2007 [42].

In poultry, diseases such as Newcastle disease and Marek’s disease have caused devastating economic losses worldwide (Table 1). Newcastle disease was regarded as one of the biggest threats to the poultry industry as this disease significantly affected poultry production throughout the world and has accounted for huge economic losses due to high mortality, high morbidity, and trade restrictions [43]. The average prevalence in adult birds was 85% in the breeding and wintering grounds of Michigan, Mississippi, and Wisconsin states of the US, and Ontario province of Canada from 2009 to 2011 [44]. The outbreak of Newcastle disease in California state of the US from 2002 to 2003, caused 3.3 million birds to be culled and cost 200 million US dollars to eradicate the virus [45]. With no effective treatment for Newcastle disease, vaccination is primarily used by the poultry industry to control the spread of disease. The multiple worldwide outbreaks of Newcastle disease in the past few years, however, have shown that the vaccination strategies are not fully effective in controlling this disease in different environmental conditions [46,47]. Marek is another disease that affects the poultry industry and is one of the most ubiquitous highly contagious viral avian infections affecting chicken flocks worldwide. Although the clinical Marek disease is not always apparent in infected flocks, the subclinical decrease in growth rate and egg production can significantly affect the economic benefits of chicken farms [48]. In Iraq, the overall prevalence of Marek disease was 49.5% with a range of 37% to 65% in different areas [49]. Even though mass vaccination is relatively efficient in controlling Marek’s disease, the appearance of highly virulent strains that can decrease vaccine immunity results in Marek’s disease virus continuing to cause a serious threat to the poultry industry [50,51]. The annual economic losses due to Marek’s disease were estimated as high as 1–2 billion US dollars worldwide [52].

As the primary source of fur among all fur industries, mink farming also suffers from the serious economic losses caused by Aleutian disease (Table 1). Aleutian disease, a chronic and persistent viral infection can cause a decrease in litter size (2.5 kits per whelping), high adult and embryonic mortalities (30–100%), and poor fur quality [53,54,55,56]. From 1998 to 2005, 24% to 71% of farmed mink were infected in Nova Scotia province of Canada [57]. The test-and-remove strategy, which is the process used to remove mink tested positive for Aleutian Disease, is employed as the main method to control Aleutian disease because of the ineffective immunoprophylaxis and treatment [58]. The unsatisfactory outcome of the test-and-remove strategy, however, makes Aleutian disease still a major problem and results in tremendous economic losses for the mink industry in North America and Europe [57,59]. The annual economic losses to the mink industry were estimated at approximately ten million US dollars in Denmark during 1984 [60].

### 2.2. Current Methods to Control Diseases in Farm Animals

Many disease-controlling methods are contributing to help farm animals cope with diseases. Vaccination, treatment, and test-based culling strategies are common approaches for the livestock industry to treat diseases and reduce the economic losses caused by subsequent health issues. Meanwhile, the development of genome editing, biosensor, and probiotics have provided more options for solving the economic and animal welfare issues caused by disease in animal farming systems. These methods have made great contributions to the control of diseases, but their deficiencies exposed in the application process cannot be ignored (Table 2).

#### 2.2.1. Vaccination

Vaccination has long been a key tool to reduce disease in livestock and maintain the health and welfare of livestock. Vaccines are contributing to preventing and mitigating many livestock diseases (e.g., Johne’s Disease and BRDC in cattle, bluetongue and sheeppox in sheep, PRRS in swine, and Newcastle and Marek’s diseases in poultry), which have complex, limited or no treatment options available, as well as reducing the use and misuse of antibiotics [79,80,81,82]. Vaccines play a significant role in preventing livestock diseases, but they also have some unsatisfactory side effects. First, vaccines are only administered to healthy subjects because they aim to prevent, not to treat. This means the vaccine can only protect the animal from disease, instead of eradication of disease [83]. Second, vaccination may cause adverse reactions in vaccinated animals. This means a vaccine may cause some adverse side effects (e.g., anaphylaxis, decrease in production traits) to a recipient [2,3]. Third, mass vaccination campaigns can be very expensive and may be unprofitable for some livestock farmers [84].

#### 2.2.2. Medical Treatments

Medical treatment is one of the main typical treatments for coping with diseases in farm animals. Veterinary drugs not only play a crucial role in controlling the diseases-related risks but also make contributions to higher agricultural productivity and a steady livestock supply [85,86]. The overall economic benefit can be increased by using the medical treatments because their applications can increase feed efficiency and performance (growth rate, egg production) for 1% to 15% more than animals that do not receive antibiotics or medical treatments [87]. Although veterinary drugs have played an important role in the field of animal husbandry and agro-industry, the increasing occurrence of residues and resistance have become issues worldwide [4,5,6,7,8,9].

#### 2.2.3. Culling

Culling infected animals and carrying strict hygiene practices are also commonly applied to control many highly contagious and inextirpable diseases in farm animals by reducing the transmission of disease. High culling rate and cost of culling make it expensive to control some diseases by culling strategy. The overall annual culling rate of 590 randomly selected dairy herds from New Zealand for BVD was 23.1% in 2002, and the cull cost for each cow was 324 US dollars [61]. About 200,000 pigs were culled from August to October of 2018 due to the outbreak of ASF in China. The direct damage from culling was estimated at about 37.8 million US dollars [88]. For controlling PRRS in Vietnam, the government needs to provide a subsidy to encourage pig farmers to voluntarily cull infected pigs [89]. This strategy, however, still cannot eradicate some of the viruses in some cases, such as Aleutian disease in mink and Johne’s disease in dairy cattle [10,11,57]. Many potential reasons such as the variability of the virus genome, ineffectiveness of biosecurity failure, viral transmission from wild animals, and persistent virus on the farms lead to the failure of culling strategies [57,90,91].

#### 2.2.4. Genome Editing

Genome editing is a powerful technology that can precisely modify the genome of an organism. The main genome editing tools are zinc-finger nucleases, transcription activator-like effector nucleases, and CRISPR/Cas9, which have been successfully employed to many farm animal species including swine, cattle, sheep, and poultry to cope with diseases at affordable costs by creating farm animals with disease-resistant genes [92,93,94,95,96,97,98,99]. There are clear opportunities especially in cases where conventional control options have shown limited success. For PRRS, the in vitro research has shown that the macrophage surface protein CD163 and specifically the scavenger receptor cysteine-rich domain 5 (SRCR5) of the CD163 protein mediate entry of PRRS virus into the host cell [100]. Based on this information, a genome-edited pig with increased resistance to PRRS virus infection could be generated with a disruption to the *CD163* gene. The genome-edited pigs created by completely knocking out the *CD163* gene [98,101] or by removing only the SRCR5-encoding genome section [102,103] showed resistance to PRRS virus infection. However, such studies did not deliver the complete resistance in the pigs in which the endogenous *CD163* gene was edited. The effectiveness of genome editing in disease control will be influenced by many factors, such as the proportion of gene-edited animals in the population and how these gene-edited animals are distributed within and across farms [96]. The disease-specific epidemiological models, however, are missing in helping with defining the exact proportion of gene-edited animals needed for each species/disease. Meanwhile, the limited shelf-life of genome editing needs to be considered. Genome editing shares the potential risk of vaccines, as the efficacy might be time-limited due to the emergence of escape mutants [96]. Especially for some RNA viruses with extremely high mutation rates, like the PRRS virus [104], this concern is justified. So far, no legal regulations have been established to supervise genome-editing animals, and all previous examples are at a preliminary stage. This means that applying this technology to farm animal production still needs a large amount of research and comprehensive monitoring systems to ensure biosafety [96]. On the other hand, public concerns about genome-edited farm animal products are also a factor that cannot be ignored, and directly determines whether genome-edited farm animal products have market value [95].

#### 2.2.5. Biosensor

A biosensor is used to quantify physiological, immunological, and behavioral responses of farm animal species through detecting specific interaction results to a change in one or more physico-chemical properties (pH change, electron transfer, mass change, heat transfer, uptake or release of gases or specific ions) [105]. This technology is applied in disease detection and isolation, and health monitoring in cattle, swine, and poultry [106,107,108,109,110,111,112]. Although the biosensor can detect abundant precise data, the data is currently not being effectively transferred into practical information that could be used for the decision-making process in farm animal health management. At the same time, the lack of investment by individual farmers has also limited the widespread application and promotion of this technology [108].

#### 2.2.6. Probiotics

The use of probiotics is also believed to have great potential to reduce the risk of the diseases of farm animals especially intestinal diseases and to replace the use of some antibiotics [113,114]. Creating a bacterial competition using probiotics, which are live microorganisms that provide a health benefit to the host when administered in adequate amounts, is a strategy to maintain health and prevent and treat infections in animals [114]. Many probiotic products are available for farm animals to improve their health and prevent them from disease [115,116,117]. Lack of statistical analysis, unclear experimental protocols, lack of precise identification of microorganisms, and missing data related to the viability of the organisms make it difficult to assess the studies associated with probiotics based on earlier research [118]. Meanwhile, the lack of an appropriate government regulatory framework and safety studies slow the industrial exploitation of novel probiotic genera and delay the large-scale application of this technology in animal farming [119].

## 3. Selection for Animals with Favorable Health Traits

### 3.1. Health Traits in Farm Animals: Definition, Classification, and Components

Historical emphasis on farm animal selective breeding programs were only focussed on profitability, and the most easily measured traits such as milk yield in dairy cows or bodyweight in swine. Recently, selection between and within breeds for health traits is attracting more attention from farm animal producers. The farmers realize that only by having a more comprehensive assessment of animal performance, the level of productivity can be maintained or improved [120].

Health traits could simply be the traits related to the health status of animals, and therefore, they could be disease traits or host immune status. According to the Animal Trait Ontology [121,122], health traits are a part of animal welfare traits. The traits could be further divided into three main groups including health body traits, disease susceptibility traits, and immune system traits. For each group, several subgroups are also included such as immune system traits which could include acquired immune system traits and innate immune systems traits. Health traits are defined by the interaction between host genetics and environment which includes the management factors as well as the pathogens. Host genetics play important roles in animals, which decide the health status of each individual in the same environment. Selection for host genetics often involves selection for disease resistance or tolerance as well as their immune systems. To maximize the host genetic potentials, it is important to study the gene by environment interaction. Genomic selection for gene by environment interaction might become more feasible using the big data [123].

Health traits could be reported at different levels as within (individual variations) or between populations. The heritabilities of health traits depend on many factors such as the nature of the traits or the method of records; however, they are known to be low-to-moderate. For instance, the estimated heritabilities for the susceptibility of cattle to Johne’s disease infection were ranged from 0.06 to 0.18 [124,125,126]. Therefore, selection for health traits can be achieved but might require quite longer time compared to the other production traits with higher heritabilities.

### 3.2. The Benefits of Selecting Farm Animals with Favorable Health Traits

**Figure 2:**
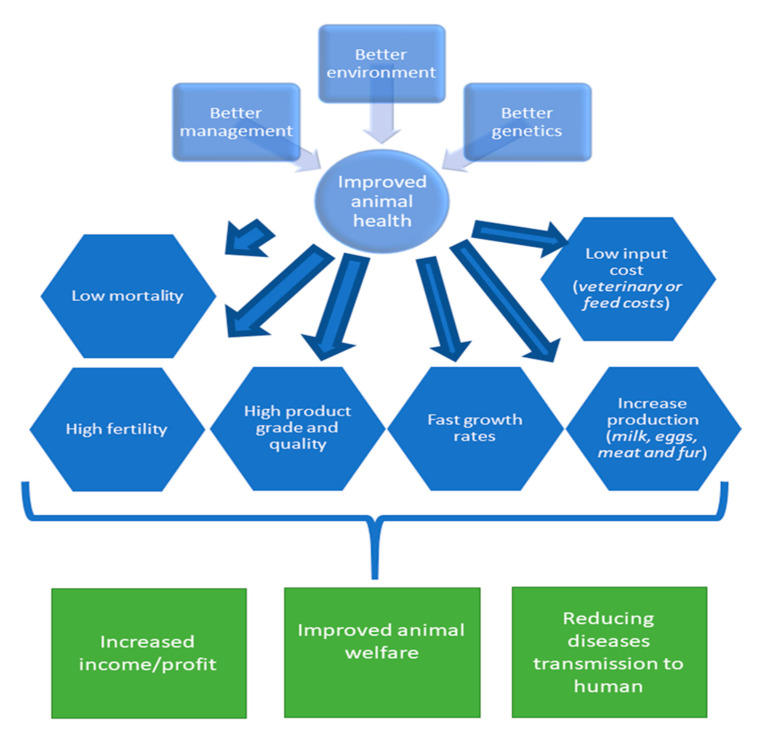
Overall benefits of selection for improved animal health.

Genetic improvement of animal health brings many benefits to the farmers, such as increase in production, reduction in the cost of treatment, enhancement of product quality and fertility (Figure 2). Overall, it improves animal welfare as less animals suffer from disease, as well as improving environmental health and human health by reducing the potential disease transmission to humans. Breeding animals with health traits for controlling disease offers several advantages over the other methods of disease control. Selecting health traits, such as disease tolerance, disease resistance, and immune response, can be an inexpensive and relatively simple way to improve animal health, welfare, and productivity. Breeding for health traits appears more and more attractive as the infectious organisms evolve resistance to the drugs and vaccines used to control them, as the costs of treatment and veterinary care increases faster than the value of the animals, and as a result of the huge economic loss caused by the culling of animals with positive disease tests results.

Protecting farm animals by vaccination or drug treatment has been the major method used to protect at-risk farm animals; however, the public concern about vaccination or drug treatment is increasing due to the drug residues and the resistance of pathogens and parasites to drugs and vaccines [127]. The intense selection pressure, which evolved into the resistance of parasites to drugs, can be imposed on the parasite population by treating farm animals with drugs such as antibiotics or anthelmintics [128]. Genetic improvement of the health of farm animals through selecting disease resistance may reduce the need for treatment with antibiotics and reduce the risk of residues in farm animal products. The worldwide control strategies to cope with helminths are entirely based on the frequent use of dewormers, which are anthelmintic drugs [129]. These control strategies have been increasingly regarded as unsustainable given the emergence of multiple drug-resistant parasites [130]. Each time an anthelmintic is employed, the resistant parasites will be selected for and will pass their resistant genes onto the next generation of worms [129]. As a result, breeding for genetic resistance is a significant component in integrated parasite management programs [131]. The genome-wide selection strategies are playing an important role in selecting animals for nematodes resistance traits [129]. The most frequent reason for using antibiotics in lactating dairy cattle is mastitis [132]. In the earlier research of bovine mastitis in Finland, the proportion of coagulase-negative Staphylococci resistant to at least one antibiotic drug increased from 27% in 1988 to 50% in 1995 and from 37% to 64% for S. aureus strains [133]. Significant increases in the antibiotics resistance were also observed in France as tetracycline resistance in *Streptococcus uberis* isolates increased from 15.7% to 20.4% and third-generation cephalosporin resistance in *Escherichia coli* isolates increased from 0.4% to 2.4% in the period from 2006 to 2016 [134]. The issues of antibiotic resistance make a permanent improvement in mastitis resistance for cow through selected breeding [135]. Vaccination can be regarded as an alternative strategy for genetic improvement of mastitis; however, a single vaccination can only provide a short-term protection instead of a permanent protection from generation to generation. Although it may be more cost-effective in the short run by using effective low-cost vaccination, genetic improvement in disease resistance has more advantages in the long run [135].

Selection for health traits can reduce the production costs associated with disease control in farm animals [136]. Culling, or test-and-remove strategy, is one of the common approaches to control highly contagious diseases such as PRRS in swine and Aleutian disease in mink. It can cause huge economic loss to farmers due to the expensive cost in replacing a diseased animal and the loss of farmed animals. Bovine tuberculosis, caused by the bacterium *Mycobacterium bovis*, is an endemic disease with zoonotic potential in many parts of the world, notably in the UK and Ireland [1]. The primary method used to control this disease is compulsory testing of cattle followed by the slaughter of test-positive animals at a total cost exceeding GBP 227 million in the UK and Ireland in 2010–2011 [137]. Highly tolerant animals still have good performance in an environment with significant virus exposure, and thus genetic selection for disease tolerance has the potential to reduce the production costs associated with culling diseased animals and eliminating the disease virus. In some developing countries, the majority of poor farmers cannot afford or do not have access to therapeutic and vaccine control, and thus the selection for healthy animals is critical for effective disease control [136].

Selection for animals with health traits (e.g., disease tolerance and disease resistance) has the potentials to bring positive economic impacts to animal farming industries. The disease-resistant animal has the ability to prevent the entry of a pathogen or inhibit the replication of the pathogen [138]. Therefore, selecting the disease-resistant animal has the potential to save the cost of medicine treatment and eliminate the economic losses caused by disease (such as reduced production, high mortality, and low fertility). The disease-tolerant animal has the ability to limit the influence of infection on its health or production performance [138]. Hence, selecting the disease-tolerant animal has the potential to minimize the adverse influence caused by disease during the production period.

### 3.3. Methods of Selection for Health Traits

Artificial selection is the process used for determining the parents for the breeding program, the number of offspring the selected parents produce, and the duration that the selected parents remain in the breeding population [139]. Artificial selection is commonly used in farm animal selection to maximize the benefits by selecting favorable characteristics and excluding the features that are not sought after by the market. The principle of selection is choosing the individuals with the best sets of alleles as genetic parents to reproduce so that the next generation has more desirable alleles than the current generation. The consequence of successful selection is genetically improving future generations of a population by increasing the proportion of desirable genes in the population over time [139]. The progress of selection for farm animal species can be viewed according to the development of molecular techniques as traditional genetic selection, marker-assisted selection and genomic selection.

#### 3.3.1. Traditional Genetic Selection

Improvement of farm animals has focused on the selective breeding of individuals with superior phenotypes. With the development of increasingly advanced statistical methods that maximize selection for genetic gain, this simple approach has been spectacularly successful in increasing the quantity of agricultural output. Selections for certain health traits have been done for a long time when the ancient people tried to select animals with better health or resistance to certain diseases during domestication [140]. These selections were purely based on their observation of performance characteristics without any information about molecular genetics. Existing selection techniques, however, still rely on laborious and time-consuming progeny-testing programs and often depend on subjective assessment of the phenotype. The traditional genetic selection breeding program evaluates the genetic potential of animals, which is based on breeding value, for some important traits using phenotype and pedigree information observed on the animal [141]. Genetic selection has significantly increased the production levels of farm animal species. The high accuracy of breeding value estimation, the moderate-to-high heritability of most production traits, and the use of large databases containing production records of many farm animal species and their genetic relationships have been found to boost breeding programs based on genetic selection and have become quite successful [142]. The application of genetic selection in commercial farm animals based on aspects of output such as higher growth rate in poultry, less fat percentage rate in swine, and greater milk yield in cows has had significant effects on outputs in the farm animal industries [143]. Genetic selection for health traits has been applied in countries with routine health data records collected for a long time. For instance, health traits have been included in breeding programs in Scandinavian countries since the mid-1970s [144]. Mastitis, ketosis and displaced abomasum diseases records were included in the breeding programs of dairy cattle in Canada [145,146]. The impacts of genetic selection for health traits depend on the nature of the traits (heritability), sample size, methods of recording, the priority of selection (e.g., economic weight in the selection index), environments and species; however, the progress for genetic selection for health traits is often lower than production traits.

#### 3.3.2. Marker-Assisted Selection

The molecular techniques such as Polymerase Chain Reaction (PCR), Fluorescence In Situ Hybridization (FISH), and Sanger sequencing were developed in the 1980s [147]. These techniques performed the amplification and sequencing of DNA and identification of markers linked to genes for economically important traits such as disease resistance. When available, these markers will provide animal breeders with an objective test system to identify the animals carrying desirable alleles at birth or even earlier such as an embryo or sperm [148]. The method allows the identification of genes or DNA markers for genetically engineering disease resistance and selection of enhanced production traits [148]. Quantitative trait loci (QTL) mapping is the first step to detect chromosomal regions affecting complex traits, which will be used in the fine mapping for identification of DNA markers for traits of interest. The QTL detection experiments in farm animals started in the 1990s when Andersson et al. [149] detected a QTL for fatness on chromosome four in pigs. Many QTLs were detected initially using initial linkage maps in either crossbreds for highly divergent traits of interest, or commercial populations where half-sib families were available. In the early 1990s, QTL experiments were based on resource populations with a few hundred animals; over time resource population size has increased to thousands of animals coupled with an increasingly large number of markers. Consequently, the number of detected QTLs has also increased rapidly in different farm animal species (Table 3). While genetic markers that are linked to the QTL could be used to choose animals for selective breeding programs, the most effective markers are the functional mutations within the trait genes. For instance, the QTL identified for milk yields and components in chromosome 14 of Holstein dairy cattle is linked to the Acyl-CoA: Diacylglycerol Acyltransferase 1 (*DGAT1*) K232A Polymorphism in Sweden [150], Germany [151], Canada [152], and China [153]. Strategies to identify markers for traits and the application of these markers are described with reference to examples of loci that control a range of different traits [154]. Detection of QTLs, and genes involving the traits of interest helps to develop the marker-assisted selection programs [155]. For example, Ruane and Colleau [156] found that the application of marker-assisted selection could increase 6% to 15% of the selection response for milk production in cattle that used multiple ovulation and embryo transfer in the first six generations of selection. However, most of the detected genes and markers only explain a small proportion of phenotypic variances, and therefore, they are not effective for the selection of quantitative traits. For instance, all genetic markers of 42k genotyping panel could only explain about 11% of phenotypic variation in mortality due to Marek’s disease virus infection in layers [157].

#### 3.3.3. Genomic Selection

High-throughput genomic technologies especially high-throughput single nucleotide polymorphism (SNP) genotyping, genotype-by-sequencing, as well as the whole genome sequencing methods, have been commercially available for more than ten years. Genomic prediction/selection was the biggest change in the artificial selection of livestock species by adapting high-throughput genotyping technologies in the farm animal sector [158]. Genomic selection refers to making breeding decisions based on genomic estimated breeding values (GEBVs) obtained from SNP effects using various prediction methods [158]. The main approach for genomic selection is to determine the SNP effects from a reference population consisting of a subset of animals with both SNP genotypes and phenotypes for traits of interest, then to use the SNP effects to compute the breeding values (genetic merit) for other genotyped animals that are not yet phenotyped. The basic statistical method used for genomic prediction is similar to the traditional best linear unbiased prediction (BLUP) method that has traditionally been used in animal breeding for a long time, except that the relationship matrix is computed based on SNP genotypes or genomic information. The major advantages of genomic selection are the higher prediction accuracy (compared to traditional EBVs obtained using pedigree information) and the shorter generation interval [159]. The accuracy of GEBVs depends on the size of the reference population used to derive prediction equations, the heritability of the trait, the extent of relationships between selection candidates and the reference population, the relationship between test and reference populations, number of SNPs, number of loci affecting the traits as well as how close assumptions in genomic prediction methods are to the truth [160,161]. Genomic selection has been successfully applied in the farm animal sections and has accelerated the genetic gain not only for the production traits but also for many health traits [162].

### 3.4. Selection for Different Types of Health Traits

#### 3.4.1. Selection for Disease Response Traits (Resistance, Tolerance, and Resilience)

Disease tolerance and resistance are the most common targeted disease response traits in farm animal breeding programs, as they are natural and distinct mechanisms of a host’s response to infectious pathogens and could be targeted for genetic improvement [13]. Resistance is the ability of a host to prevent the entry of a pathogen or inhibit the replication of the pathogen. Tolerance is an ability of a host to limit the influence of an infection on the host’s health or production performance without interfering with the life cycle of the pathogen [138].

To date, most efforts to control infectious disease focus on selecting disease resistance farm animals to improve the ability of the host to fight disease. The heritable differences of disease resistance between animals lead to opportunities to breed animals for enhanced resistance to the disease [163]. In cattle, the major focus on health traits selection is for mastitis resistance. Many different approaches have been proposed in order to increase the possibility of selection for mastitis [164]. Up to date, 2382 QTLs have been identified for mastitis resistance in dairy cows (Animal QTL Database, https://www.animalgenome.org/cgi-bin/QTLdb/BT/nscape?isID=1439). Not only increasing the number of QTLs, the genetic and genomic selection for mastitis has also achieved a certain level of success (reviewed by Weigel and Shook, [165]) because of the increasing accuracy of prediction for mastitis or the inclusion of different new methods of identification of mastitis incidence in the selection index. For instance, the accuracy of genomic prediction could reach as high as 0.50 to 0.55 for mastitis infection depending on the models [166]. Unlike mastitis, less progress is reported for selection for Johne’s disease and BRDC resistance, which might be due to the lack of accurate measurements and their less serious impact on production. The heritabilities for Johne’s disease (range from 0.07 to 0.16) and BRDC (range from 0.07 to 0.19) resistance and differences among breeds have been documented in the previous studies [20,124,167,168]. These heritability estimates and significant estimates of additive genetic variances indicate that computing traditional phenotype-based genetic evaluations for resistance to Johne’s disease and BRDC is feasible in cattle populations. In swine, 43 QTLs for PRRS resistance have been mapped to 12 chromosomes (Animal QTL Database, https://www.animalgenome.org/cgi-bin/QTLdb/SS/traitmap?trait_ID=779). The major QTL region was located on chromosome four (SSC4) that explained 16% of the genetic variance of PRRS virus load with a frequency for the favorable allele of 0.16 and a heritability of 0.30 [169]. In poultry, a number of QTLs associated with Marek’s disease resistance have been reported in various lines and breeds of chicken using SNP or microsatellite markers since 1998 [170,171,172,173,174].

The research focus associated with selecting health traits has expanded to increase the host’s tolerance to reduce the harmful effects of infection on health and performance [13,175]. Genetic selections of disease tolerance are rare, as the genetics of disease tolerance and its measurement are more difficult to elucidate than disease resistance in farm animals [1,176]. Growing evidence, however, indicates the potential for genomic selection of disease tolerance. Genomic studies have been able to map the QTL for tolerance traits as Zanella et al. [177] identified a number of QTLs for Johne’s disease and Hanotte et al. [178] detected 16 QTLs for trypanosomosis, in the cross of N’Dama and Boran cattle. Meanwhile, the results of genomic prediction (accuracy of 0.38) for facial eczema suggested that genomic selection for the facial eczema disease tolerance has the potential to help the New Zealand sheep industry to cope with the issues caused by facial eczema [179].

Although both resistance and tolerance traits may be under genetic control and could thus be targeted for genetic improvement, selecting tolerance for disease may have some advantages over selecting disease resistance [176]. Firstly, the resistance ability of a host can limit the replication of a pathogen within the host, and therefore, selecting host resistance has a potential to increase the selection advantages on pathogen strains that can withstand host resistance mechanisms and eventually result in a loss of selection advantage of the host [180,181]. It is the potential pitfall for a long-term breeding strategy which focuses on disease resistance if the disease virus has a high mutation rate such as the PRRS virus in swine [182]. It has been theoretically proposed that selecting tolerance might not motivate such selection pressure on the pathogen [181]. Secondly, compared with the resistance mechanisms which directly influence the life-cycle of the pathogen, improving host tolerance has the potential to provide cross-protection against other strains of the virus, or other prevalent infectious agents due to the mechanisms of tolerance which primarily target host-intrinsic damage prevention or repair mechanisms [175,183,184].

Resilience is another health trait that is attracting the attention of animal breeders. Generally, resilience is an ability of an animal either to minimize the influences caused by disturbances or to return to the body condition prior to exposure of a disturbance [185]. The capability of taking care of a larger number of animals is one of the requirements for the intensification of farm animal production. Selecting resilient animals can improve this capability of the farm animal industries because resilient animals are healthy and easy-to-care-for animals that need less attention time [186]. On the other hand, compared to the direct selection based on disease tolerance and resistance, the selection based on resilience is a more pragmatic way of keeping healthy animals, because it does not need the records on pathogen burden, which is the amount of pathogen in the animal’s body [187,188,189]. Resilience, however, is not yet included in breeding goals due to the difficulty of phenotyping [13]. Fortunately, the current developments on the big data collection and new disease resilience indicators defined based on these data provide great opportunities to breed for improved resilience in livestock [190].

#### 3.4.2. Selections for Immune Response Traits

Immunity response traits are also important health traits for animal breeders to select for improving the farm animals’ ability to withstand disease. The immune system is important to control infections and diseases. The immune response traits have been recommended to be selected for decreasing the incidence and impact of the disease in farm animals [14,191]. In Holstein cattle, the lower occurrence of mastitis improved response to the commercial vaccine, and increased milk and colostrum quality are all observed in cows with superior or high immunity response [118]. Consequently, improving the inherent ability to cope with the diseases in dairy cattle through genetic selection for superior or high immunity response is feasible [192]. In cattle, the High Immune Response (HIR™) and the Immunity+, which are used to identify and select animals with naturally optimized immune responses, have been applied in the genetic selection of cattle for improved immunity and health [14]. In swine, the total and differential numbers of leukocytes, expression levels of swine leukocyte antigens I and II, and serum concentrations of IgG and haptoglobin are immunity traits that have been demonstrated to have additive genetic variation. These immunity traits, therefore, have the potential to be used as criteria to improve the selection of pigs for coping with clinical and subclinical diseases [193]. In poultry, the presence of genetic variability in immune response traits and the discovery of SNPs associated with immune response traits indicate that genetically enhancing antibody response and resistance to parasitism is feasible through genomic selection [194].

### 3.5. Challenges in the Selection of Health Traits

Health traits, such as disease resistance, disease tolerance, and immunity response level are usually quantitative traits which are influenced by many genetic and environmental factors. Although genetic selection has significantly increased the production traits in farm animal species such as higher growth rate, less fatness, and greater milk yield [143], selection for health traits is much more complicated and faces some challenging obstacles. The potential problems in selection for health traits can be classified under desirability, feasibility and sustainability [195].

#### 3.5.1. Desirability

The desirability describes the importance of the disease relative to the other diseases or production traits. The correlations between health traits and economic traits are often negative, which means the health traits are potentially genetically antagonistic to production traits [196,197]. Milk yield in dairy cattle has unfavorable correlations with many disease response traits [198,199]. The genetic correlations between mastitis and milk production or high somatic cell score and milk production are moderate and positive [200]. In poultry, genetic selection for greater body weight can lead to decreased immunity to fowl cholera and Newcastle disease [201]. The opposite results, however, also occur in some research. For example, van der Most et al. [202] stated that selection for growth in poultry can compromise the immune function, while the selection for immune function does not consistently affect growth. Therefore, identifying the genetic correlations between health traits and production traits in farm animals is an important aspect of health traits selection. Applying the economic selection index is one of the solutions to deal with the antagonistic genetic correlation between traits. In 1943, Hazel [203] first presented the aggregate genotype, which was also called net merit of animals as a linear combination of breeding values for each trait weighted by the economic value of the traits. After that, the economic selection index for multi-trait selection has been used in animal breeding research fields and employed in animal agriculture industries. The breeding objective can be defined as the aggregate breeding value expressed by profit or economic efficiency, and it is the overall goal of breeding programs to increase the profits or economic efficiency for breeders and/or producers. In this way, multi-trait selection with the economic selection index can minimize the adverse influences caused by the antagonistic genetic correlations between target traits to achieve the overall goal of breeding programs [204].

#### 3.5.2. Feasibility

Feasibility accounts for the tools available with which to perform the selection. The success of selection for health traits is highly dependent on correctly identifying the phenotype for traits associated with the host’s abilities to withstand infectious diseases. Accurately identifying the phenotypes for health traits is expensive and difficult. An extensive data recording is required to enable an accurate genetic evaluation. High labor costs are required for long-term recording of large amounts of phenotypic and progeny data [12]. In a combined population of infected and healthy individuals, it is not correct to consider an individual with good performance to have favorable health traits, nor the sick populations to be genetically susceptible [205]. Some susceptible animals still show good performance because they may not have been sufficiently exposed to the pathogens. An animal displaying a healthy performance without clinical symptoms may have sub-clinical infections and represents a pathogen carrier. The clinical expression of a disease can be confounded by infection with one or more similar diseases such as pneumonia which can be confused with pulmonary adenomatosis, bronchitis, and pleuritis. Meanwhile, diagnosing a disease accurately and specifically is costly and time-consuming [196].

#### 3.5.3. Sustainability

Sustainability means the enhanced resistance to the infectious disease in the farms or flocks is stable for a long period especially when the pathogens often evolve faster than the hosts [195]. The long-term success of selection involves not only the choice of the best animals with disease resistance but also the management systems with the ability to cope with the constant changes in the farming environment. For instance, hot environment caused by global warming could impair production and reproductive performance, metabolic and health status, and immune response [206]. The climate changes also cause changes in the pathogens or create novel pathogens which require the producers to constantly adapt new methods and treatments for their animals. Genomic selection of robustness and fitness traits could be a solution for this challenge [190,207].

### 3.6. Promise of Selection for Health Traits

#### 3.6.1. High-Throughput Phenotyping and Sequencing, and Generation of Big Data

Big data is a mix of different sources of data (structured and unstructured) that comprises a large volume of information [208]. The major characteristics of big data include volume, velocity, variety, variability, veracity, validity, and volatility [209]. Big data has been adapted to the farm animal sector such as precision farming [210], biosensors [211], electronic feeding stations, and automatic milking systems [123]. Big data is also important for infectious disease surveillance and modeling [190,212]. It is clear that big data generated from high-throughput phenotyping will give unprecedented opportunities for combating diseases and selecting healthy animals [213,214]. For example, the mastitis and claw health can be recorded via high-throughput phenotyping devices such as real-time biosensors [215,216]. The use of big data for animal health care, however, needs a careful handling of the data [217] and selection of appropriate statistical methods [218,219]. High-throughput sequencing data, such as genomics, transcriptomics, proteomics, and epigenomics etc., have been adapted to improve animal health [220,221] as they could help to understand the biology of disease, computing EBVs, and pinpointing the biomarkers.

#### 3.6.2. Data Sharing and International Corporations

Data sharing and international corporations can play crucial roles in the selection of healthy traits even those selections that take place locally. The major reason for this is that many diseases in farm animals are transboundary diseases. The outbreaks of diseases could potentially affect other farms in different countries such as the outbreaks of Avian Influenzas Virus that cause significant loss in many nations worldwide. Information sharing plays a crucial role in controlling diseases for nations on the same continent especially for developing countries [222]. It is also important to have a standard protocol for recoding the incidences, progress of the disease and consequences of diseases for better use of data. In cattle, for instance, the International Committee for Animal Recording provides a recording guideline for 1000 diagnoses that can be used toward the genetic improvement of health traits (ICAR GUIDELINES, https://www.icar.org/index.php/icar-recording-guidelines/). International corporations could work together in a joint effort for phenotyping or genotyping animals/disease to enlarge the resources and enhance the human capacity to deal with disease. For example, the use of automatic milking systems from different nations could improve the modeling of mastitis infections [165] or the sharing of omics data could better develop the statistical methods and enhance understanding about the disease biology [223]. The current 1000 Bull Genomes Project is a successful story regarding the sharing of genomic data for improving the prediction accuracy of future genomic EBVs [224]. It is important to indicate that increasing the capacity of cloud storage and computing could also support the sharing of data and corporations.

## 4. Conclusions

Selecting favorable health traits to cope with diseases in farm animals has increasingly become an attractive focus of animal farming industries. Given some limitations and deficiencies of current non-selection disease control methods and the advantages of genetic selection over the other methods, breeding for health traits is a promising solution for the sustainable development of livestock farming. Although some remaining challenges regarding the accuracy of phenotyping and low heritability of disease traits hinder the progress of breeding for health traits, the advancement of sequencing techniques and affordable cost of genotyping make selective breeding more beneficial as a method for disease control but also require more storage and computing power. With the development of cloud computing, big data analyses increase the feasibility of selection for animal health traits. Increasing threats, such as climate change, have caused changes in the environments that require international collaborations to deal with the disease on a global scale. Eventually, smart farming with healthy animals and clean environments will be achieved with the sustainable selection methods of favorable health traits. The genetic and genomic selection solution, however, cannot address all the problems caused by disease farm animals. Therefore, it is necessary to accompany selection solution approaches with other disease control and monitor methods (e.g., vaccination, culling strategy, biosensor, and genome editing) to help animal agriculture industries to reduce the economic losses and animal welfare issues caused by farm animal diseases.

## Figures and Tables

**Table 1:** Prevalence and economic losses of common diseases and their impacts on performance in farm animal species.

Species	Disease	Prevalence	Economic Losses	Milk Yield	Fertility/Egg Production	Growth Rate	Mortality	Vaccine Available?	Specific Treatment?
Cattle	Bovine Viral Diarrhea	Up to 98.5% and 98.3% in non-vaccinated dairy and beef herds, respectively [15]	40–100 thousand USD per herd in Canada [16] and 10–40 million USD per million calvings in Europe [17]	Reduced (~0.074 kg/day [61])	Reduced (21% abortion rate [62])	Reduced	High (~50% in calves [63])	Yes	No
	Johne’s Disease	68.1% of US dairy operations were infected [20]	15 million CAD annually in Canada and 200–250 million USD in US [21]	Reduced (up to 25% [64])	Reduced (7% lower rate of conception [65])	Reduced	Culling infected individuals	Yes	No
	Bovine Respiratory Disease Complex	45.9% in UK dairy heifers	One billion USD annually in US [24]	N/A	N/A	Reduced	Moderate (~20% in calves [66])	Yes	No
Sheep	Bluetongue	19% in Italy [26] and up to 94.3% in Sudan [27]	In 2007, 12.6 million euros in the Netherlands [28]	Reduced (up to 42% [67])	Reduced (25% abortion rate and 50% decrease in fertility [68])	Reduced	High (up to 41.5% [69])	Yes	No
	Sheeppox	Up to 22% [31] in India and 40% in Ethiopia [32]	2.4 million USD annually in Maharashtra, India [33]	N/A	N/A	Reduced	High (up to 40% [70])	Yes	No
Swine	Porcine Reproductive and Respiratory Syndrome	Up to 48% of pig farms in Ontario, Canada [35]	664 million USD annually in US [36] and 130 million CAD annually in Canada [37]	N/A	Reduced (up to 40% abortion rate [71])	Reduced	High (up to 100% [72])	Yes	No
	African Swine Fever	12.5% in China from August 2018 to July 2019 [41]	1.25 billion USD from 2007 to 2017 in Russia [42]	N/A	Reduced (54% abortion rate [73])	N/A	High (30–70% [74])	No	No
Poultry	Newcastle Disease	85.2% in eastern North America between 2009 and 2011 [44]	200 million USD from 2002 to 2003 in California, US [45]	N/A	Reduced (55% of egg production [75])	Reduced	High (up to 100% [76])	Yes	No
	Marek’s Disease	49.5% in Iraq [49]	1–2 billion USD annually worldwide [52]	N/A	Reduced (decrease 5% egg production [77])	Reduced	Moderate (10–30% [78])	Yes	No
Mink	Aleutian Disease	Up to 71% in Nova Scotia, Canada between 1998 and 2005 [57]	10 million USD in Denmark during 1984 [57]	N/A	Reduced fertility (~2.5 kits per whelping [56])	Reduced	High (30–100% [55])	No	No

**Table 2:** Strengths and weaknesses of common non-selection disease control methods in farm animals.

Controlling Method	Advantages	Disadvantages
Vaccination	▪ Prevent and mitigate various diseases in livestock ▪ Provide solutions to control diseases which have complex, limited or no treatment options available ▪ Decrease the antimicrobial resistance	▪ Only administered to healthy subjectsMay cause adverse reactionsBring expensive cost for large-scale use
Medical treatment	▪ Treat many common diseases in livestock species ▪ Increase in feed efficiency and performance	▪ Increase the occurrence of drug residues ▪ Increase the risk of drug resistance
Culling	▪ Main method used to control highly contagious and inextirpable diseases	▪ Fail in permanently eradicating some diseases from livestock farms ▪ High reinfection rate in some cases ▪ Very costly in large-scale farms
Genome editing	▪ Offer solutions to control untreatable diseases at affordable costs ▪ Has high efficiency and low cost in controlling diseases	▪ No legal regulations have been established to supervise genome-editing animals ▪ Is not mature enough for large-scale use ▪ Public’s concerns
Biosensor	▪ Effective in disease detection and isolation, and health monitoring	▪ Not effective in practical livestock health management ▪ Not widespread and promoted due to the lack of investment
Probiotics	▪ Have great potential to reduce the risk of intestinal diseases ▪ Have the potential to replace some antibiotics	▪ Lacking adequate related research ▪ Unable to apply in large-scale livestock farming

**Table 3:** The number of quantitative trait loci (QTLs) detected in animal species by 7 July 2020.

Species	Number of Publications	Number of Traits	Overall	Health	Disease Suppressibility	Immune Capacity	Pathogens and Parasites	Blood Parameters
Cattle	1001	646	142,261	6380	2771	232	124	355
Chicken	328	430	12,246	820	739	NA	NA	294
Horse	94	56	2446	1128	1026	19	NA	1
Swine	698	691	30,580	6598	586	3230	81	2747
Sheep	173	262	3305	619	135	39	335	37
